# Monocyte subsets in breast cancer patients under treatment with aromatase inhibitor and mucin-1 cancer vaccine

**DOI:** 10.1186/s12967-024-05659-w

**Published:** 2024-10-08

**Authors:** Viktoria Knöbl, Lukas Maier, Stefan Grasl, Carmen Kratzer, Felix Winkler, Vanessa Eder, Hubert Hayden, Maria Amparo Sahagun Cortez, Monika Sachet, Rudolf Oehler, Sophie Frantal, Christian Fesl, Karin Zehetner, Georg Pfeiler, Rupert Bartsch, Florian Fitzal, Christian F. Singer, Martin Filipits, Michael Gnant, Christine Brostjan

**Affiliations:** 1 Division of Vascular Surgery, Department of General Surgery, Medical University of Vienna, University Hospital Vienna, Vienna, Austria; 2 Division of Visceral Surgery, Department of General Surgery, Medical University of Vienna, University Hospital Vienna, Vienna, Austria; 3 Comprehensive Cancer Center, Medical University of Vienna, Vienna, Austria; 4 Austrian Breast & Colorectal Cancer Study Group (ABCSG), Vienna, Austria; 5 Department of Obstetrics and Gynecology, Medical University of Vienna, University Hospital Vienna, Vienna, Austria; 6 Division of Oncology, Department of Medicine I, Medical University of Vienna, Vienna, Austria; 7 Department of General Surgery, Hanusch Hospital, Vienna, Austria; 8 Center for Cancer Research, Medical University of Vienna, Vienna, Austria

**Keywords:** Aromatase inhibitor, Breast cancer, Estrogen receptor, Letrozole, Monocyte, Stimuvax, Tecemotide

## Abstract

**Background:**

Monocytes comprise subsets of classical, intermediate and non-classical monocytes with distinct anti- or pro-tumor effects in breast cancer (BC). They are modulated by estrogen, and can contribute to BC control by endocrine therapy in preclinical models.

**Methods:**

To elucidate whether changes in monocyte subsets are associated with treatment and response, we investigated peripheral blood samples of 73 postmenopausal women with estrogen receptor (ER) positive BC, who received aromatase inhibitor therapy with or without the mucin-1 vaccine tecemotide in the ABCSG34 trial. Blood was retrieved at baseline, midterm and end of therapy, and was analyzed for the distribution and ER expression of monocyte subsets by flow cytometry.

**Results:**

When 40 healthy, age-matched women were compared with BC patients before treatment start, ER levels of monocytes did not differ, yet patients presented with a higher frequency of classical and fewer non-classical monocytes. Endocrine therapy triggered a significant increase in ER levels in all monocyte subsets, without affecting subset distribution. Vaccination had no overall impact on subset frequency and ER expression. Yet, a shift from intermediate to classical monocytes during therapy correlated with changes in plasma cytokines and chemokines and was significantly associated with low residual cancer burden in vaccinated patients. Without tecemotide, baseline ER levels in classical monocytes were significantly higher in women with good response to endocrine therapy.

**Conclusions:**

This study identified classical monocytes to be associated with ER positive BC and with patient response to neoadjuvant endocrine treatment and cancer vaccination.

**Supplementary Information:**

The online version contains supplementary material available at 10.1186/s12967-024-05659-w.

## Background

Breast cancer (BC) is the most frequently diagnosed malignancy in women worldwide [1]. 80% of BC patients are women older than 50 years and menopausal [2]. In general, breast cancer is divided into 5 subtypes based on the expression or absence of estrogen receptor (ER), progesterone receptor (PR), human epidermal growth factor receptor 2 (Her2) and proliferation markers. ER positive (ER+) tumors are the most prevalent subtype with 70–75% abundance and are predominantly treated by endocrine therapy, but show substantial rates of recurrence and death [3].

Physiologically, 17β-estradiol (E2) is the major circulating estrogen in women. It triggers cellular responses mainly by binding to cytosolic ER alpha (ERα) or beta (ERβ) thereby forming a ligand-activated transcription factor which translocates to the nucleus. The ER-ligand complex either directly binds to estrogen response elements in target gene promoters or exerts indirect effects through interaction with other transcription factors and molecules [4]. Since estrogen promotes the growth of ER + tumors, BC treatment by endocrine therapy is well established and commonly based on ER modulators like tamoxifen, on aromatase inhibitors (AIs) such as letrozole, or on selective ER degraders like fulvestrant [5]. AIs have evolved as the treatment of choice for postmenopausal women, also in the setting of neoadjuvant therapy for locally advanced ER + BC, which frequently involves the third-generation AI letrozole blocking the conversion of androgen to estrogen [6].

Several preclinical studies suggested that AIs might be suited to sensitize ER + tumors to the effects of a cancer vaccine directed against mucin-1 [7–9]. The transmembrane glycoprotein mucin-1 (MUC1 or CD227) and soluble mucin-1 (CA15-3) is generally expressed by epithelial cells, but is overexpressed and aberrantly glycosylated in adenocarcinomas including 80–90% of breast cancers [10]. It promotes tumor growth by anti-apoptotic, anti-adhesive and immunosuppressive effects [11–13]. In the Austrian Breast & Colorectal Cancer Study Group (ABCSG) trial 34 the combination of neoadjuvant treatment by letrozole and the MUC1 vaccine tecemotide was recently investigated for postmenopausal women with non-metastasized, ER+, Her2- BC [14–17]. The synthetic lipopeptide tecemotide is a MUC1-specific antigen which was anchored in a liposomal membrane (liposomal BLP25; L-BLP25; Stimuvax^®^) designed to trigger cytotoxic T-cell responses against MUC1-expressing cancer cells [8, 9]. The addition of tecemotide to endocrine therapy increased patient response from 13% (letrozole) to 25% (letrozole and tecemotide) in the ABCSG34 trial, as defined by residual cancer burden (RCB) which was, however, not statistically significant (*p* = 0.17) [15].

Although tecemotide was primarily devised to drive adaptive anti-tumor responses, it may also affect mediators of the innate immune system including monocytes. MUC1 is expressed on the surface of healthy human monocytes [18] and the aberrantly glycosylated tumor-derived MUC1 was found to interact with monocytes and drive them to an immunosuppressive state [19, 20]. Furthermore, monocytes are responsive to estrogen and therefore influenced by endocrine therapy [21]. As shown in numerous in vitro studies, the effects of estrogen on human monocytes are primarily repressive, in particular with respect to pro-inflammatory responses by the release of cytokines [22–24], by chemotaxis and adhesion [25–27]. Estrogen also affects monocyte proliferation and survival [28, 29]. Thus, endocrine therapy might indirectly support cancer control by relieving the repressive effects of estrogen on monocytes. A preclinical study by Iyer et al. presented evidence that not only ER + but also ER- BC growth is promoted by estrogen via its mobilizing and activating impact on a pro-angiogenic monocyte subset [30].

Of note, human monocytes constitute a heterogeneous cell population with distinct subsets categorized into classical, intermediate and non-classical monocytes [31]. The detection of these subpopulations is based on the surface markers CD14 and CD16, with classical (CD14 + + CD16-), intermediate (CD14 + + CD16+) and non-classical (CD14 + CD16++) monocytes exerting distinct and partly opposing functions in cancer biology. In particular, the CD16 + monocyte subsets are considered tumor-promoting by their tissue-remodeling, angiogenic activities and their favorable cytokine profile [32]. When we previously characterized features of monocyte subsets isolated from healthy individuals as well as cancer patients [33] we found a remarkably consistent expression pattern which was in line with other literature [34, 35]: Classical monocytes presented with highest CD36 levels (related to scavenging and phagocytosis), while the non-classical subset was the predominant producer of tumor necrosis factor alpha (TNFα) and the intermediate monocytes were characterized by highest expression of C-C chemokine receptor 5 (CCR5) and HLA-DR (for antigen presentation).

In view of the differential involvement of monocyte populations in BC and their potential regulation by endocrine therapy as well as by tecemotide we hypothesized that the frequency of monocyte subsets and their ER expression might be altered during cancer therapy, possibly indicating patient response to treatment. We therefore monitored the distribution of classical, intermediate and non-classical monocytes in peripheral blood of BC patients and determined the intracellular levels of ERα in monocyte subsets in the context of the ABCSG34 trial.

## Methods

### Study design

The ABCSG34 trial was designed as a prospective, multicenter, randomized phase II study evaluating tecemotide as component of neoadjuvant therapy in women with histologically proven, invasive, Her2 negative, non-metastasized BC who were scheduled to receive neoadjuvant endocrine therapy or third-generation chemotherapy. The endocrine treatment cohort comprised postmenopausal women with tumors defined by either high estrogen receptor expression (ER+++) or by intermediate ER + + levels combined with a Ki67 proliferation rate lower than 14%. Further inclusion criteria were tumor grade 1, 2 or X (unassessable) of patients aged ≥ 18 years with a WHO performance status of 0 or 1 (and adequate hematologic, renal, hepatic and cardiac function) who were scheduled for endocrine therapy as standard of care. Clinical exclusion criteria comprised the following: HER2 overexpression, prior chemotherapy, radiotherapy, or endocrine therapy for invasive breast cancer, past or current history of other malign neoplasms in the last 5 years (except basal cell cancer of the skin, non-melanoma skin cancer and in situ cancer of the cervix), concurrent or prior systemic antitumor therapy < 5 years, clinically significant cardiovascular disease, autoimmune disease or compromised hematopoietic function or known immunodeficiency, any uncontrolled infections < 14 days prior to randomization, positive tests for human immunodeficiency virus infection, hepatitis C virus, acute or chronic active hepatitis B infection, major surgery or significant traumatic injury occurring within 4 weeks prior to randomization, any other severe acute or chronic medical or psychiatric condition, known hypersensitivity against aromatase inhibitors, concurrent treatment with corticosteroids except as use for prevention or treatment of acute hypersensitivity reactions.

**Fig. 1:**
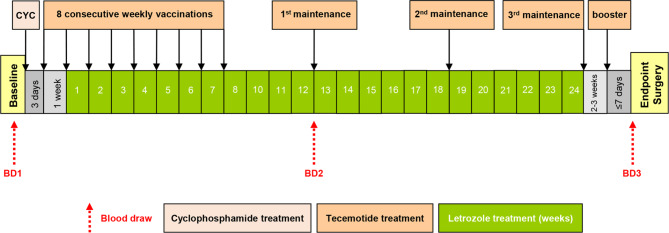
Schematic representation of the treatment schedule for BC patients receiving letrozole-based endocrine therapy with or without additional cyclophosphamide (CYC) administration and tecemotide vaccinations. Three blood draws (BD1-3) served to assess monocyte populations over the course of neoadjuvant therapy

Patients were randomized 1:1 to either receive endocrine treatment or endocrine treatment plus tecemotide. The AI letrozole was given as standard-of-care at 2.5 mg (*per os* once per week) for 24 consecutive weeks. Patients in the tecemotide arm additionally received one intravenous infusion of cyclophosphamide (300 mg/m^2^) 3 days before starting vaccination to possibly increase the efficacy of immunotherapy. This was followed by 8 consecutive weekly vaccinations of 930 µg tecemotide, 3 maintenance shots at weeks 12, 18, and 24, and a final booster vaccination 2–3 weeks after the last maintenance shot. Blood samples were retrieved at baseline/screening, mid-therapy after 12 weeks of AI treatment and at the end of neoadjuvant therapy, i.e. prior to surgery (Fig. 1).

Primary endpoint of the clinical study was efficacy of neoadjuvant treatment as assessed by residual cancer burden (RCB) according to Symmans [36] which includes the cellularity of the invasive tumor component, the primary tumor bed dimensions, and the axillary lymph node burden. RCB scores were categorized as no (RCB 0) or minimal residual disease (RCB I ≤ 1.36) as opposed to more extensive residual cancer burden (RCB > 1.36).

The study was conducted according to the Declaration of Helsinki and the ICH Guidelines (EudraCT no. 2011-004822-85) and was approved by the ethics committees of the 17 involved clinical centers. Forty age-matched healthy women were additionally recruited as a control cohort to compare baseline levels of monocyte subsets and ER expression levels between BC patients and non-tumor bearing women (approval by the ethics committee of the Medical University of Vienna: #331/2010 and #925/2011). Patients and controls gave written informed consent.

### Monocyte analysis by flow cytometry

In total, 30 ml of peripheral venous blood was drawn from each patient per time point of which 24 ml were collected in Vacutainer CPT Tubes (Cell Preparation Tubes by Becton-Dickinson, Franklin Lakes, NJ, USA) for peripheral blood mononuclear cell (PBMC) isolation, 3 ml in CTAD (citrate, theophylline, adenosine, dipyridamole) containing tubes for plasma preparation and 3 ml in EDTA (ethylenediamine tetraacetic acid) tubes - both by Greiner Bio-One, Kremsmünster, Austria - for whole blood staining and flow cytometry. Samples were transported at room temperature and processed within 24 h. Since whole blood samples were found particularly stable over 24 h with respect to the detection of monocyte subset distribution, preference was given to immunostaining and flow cytometry of whole blood samples rather than isolated PBMCs [37].

Thus, the frequency of monocyte subsets was determined by immunostaining of 50 µl EDTA-anticoagulated whole blood with 5 µl HLA-DR-APC/Cy7 (no. 307618, BioLegend, San Diego, CA, USA), 10 µl CD14-FITC (no. 555397, Becton-Dickinson) and 5 µl CD16-PC5 (no. A07767, Beckman Coulter, Brea, CA, USA) antibodies at room temperature (RT) for 20 min. After erythrocyte lysis by the addition of 500 µl Versa Lyse (no. A09777, Beckman Coulter) and incubation for another 20 min at RT, monocytes were analyzed with a Gallios™ flow cytometer and Kaluza 2.1 software (Beckman Coulter). Monocytes were defined as CD14 and HLA-DR positive, with further distinction between classical (CD14 + + CD16-), intermediate (CD14 + + CD16+) and non-classical (CD14 + CD16++) monocytes as previously reported [38] and illustrated in Supplementary Fig. 1.

To detect the intracellular ER expression level of monocyte subsets, samples stained for the surface markers HLA-DR, CD14 and CD16 were fixed and permeabilized with IntraPrep™ Permeabilization Reagent Kit (Beckman Coulter) according to manufacturer´s instructions: EDTA-anticoagulated whole blood was 1:2 diluted with phosphate-buffered saline (PBS) and then distributed on two sample tubes at 50 µl each. After addition of 5 µl HLA-DR-APC/Cy7, 10 µl CD14-FITC and 5 µl CD16-PC5 antibodies and incubation at RT for 15 min, blood cells were fixed with 100 µl reagent 1 of IntraPrep Kit for 15 min at RT. Following sample dilution with 3.5 ml PBS, a centrifugation step was conducted for 5 min at 300 x g and RT. The pellet was carefully resuspended in 100 µl reagent 2 of IntraPrep Kit and incubated for 5 min at RT for cell permeabilization. The two sample aliquots were now separately labeled for 15 min at RT with either 5 µl of biotinylated anti-ER Ab-1 (clone AER314, no. MS-168-B1 by ThermoFisher Scientific, Waltham, MA, USA) or 5 µl of corresponding isotype control mIgG_1_-biotin (no. IM3036, Beckman Coulter). After sample dilution with 3.5 ml PBS and another centrifugation step for 5 min at 300 x g and RT, the pellet was again resuspended in 100 µl reagent 2 for 5 min at RT. Finally, 2 µl of streptavidin-PE conjugate (no. STAR4A, AbD Serotec, Bio-Rad, Hercules, CA, USA) were added for 15 min at RT, followed by another PBS dilution and centrifugation step before pellet resuspension in 500 µl cold (4 ^o^C) PBS buffer containing 2.5% fetal bovine serum (FBS) and 0.25% formaldehyde. Monocyte subsets were then analyzed for intracellular ER expression by Gallios™ flow cytometer as illustrated in Supplementary Fig. 1.

### Analysis of plasma cytokines by multiplex immunoassay

For plasma preparation, CTAD-tubes were subjected to an initial centrifugation step at 1000 x g and 4 °C for 10 min. The plasma supernatant was collected and further centrifuged at 10,000 x g and 4 °C for 10 min to remove remaining platelets. The supernatant was stored in aliquots at -80 °C. Samples were analyzed by Olink Bioscience (Uppsala, Sweden) with Proseek Multiplex Inflammation I Immunoassay for the simultaneous detection of 92 human biomarkers: 78 of 92 of parameters were detected in > 20% of samples and 99% of the assays had an intra CV < 10%.

### PBMC isolation

PBMCs of BC patients were isolated from heparin-ficoll containing CPT sample tubes (no. 362780, Becton-Dickinson). Within 1 h of blood withdrawal, CPT blood tubes were centrifuged for 20 min at 1650 x g and RT to separate PBMCs by an inert gel barrier. After sample transport to the laboratory within 24 h, the PBMC-plasma fractions of 24 ml patient blood were harvested from the CPT tubes and centrifuged again for 15 min at 350 x g and 10 °C. The cell pellet was resuspended in 50 ml PBS and subjected to another round of centrifugation for 15 min at 350 x g and 10 °C. A second washing step with 6 ml PBS and centrifugation was conducted. PBMCs were cryopreserved at about 10 × 10^6^ cells per ml (of pre-chilled heat-inactivated FBS containing 5% dimethyl sulfoxide) in a freezing container at -80 °C over night, and were finally stored in liquid nitrogen. When tested, the isolated PBMCs had few dead cells and a mean percentage of 18.7 (SD 8.9%) monocytes. On average, 71.2% (SD 3.0%) of PBMCs survived the freezing/thawing process including a fraction of 15.4% (SD 4.6%) monocytes.

### Subcellular ER analysis in patient PBMCs by confocal microscopy

1 ml of PBMCs were thawed and centrifuged in 20 ml RPMI 1640 medium without phenol red (Lonza, Basel, Switzerland) supplemented with 10% heat-inactivated FBS for 7 min at 300 x g and RT. Cells were resuspended in 1 ml medium, counted with a Sysmex XN-350 device (Sysmex, Kobe, Japan) and adjusted to 5 × 10^5^ cells per ml. 25,000 cells in a total volume of 50 µl medium were spun onto a glass slide at 30 x g at RT for 7 min (Rotofix 32 A, Hettich, Tuttlingen, Germany). Cytospins were dried for 30 min, then fixed with ice-cold methanol for 10 min and stored at 4 °C until immunofluorescence staining.

Cytospins were washed twice for 5 min in PBS without calcium and magnesium (PBS-/-). Cells were then permeabilized with 0.1% Triton X-100 in PBS-/- for 10 min at RT followed by three washes with PBS-/- (of 5 min each). Slides were blocked for 60 min at RT in PBS-/- with 1.5% BSA and 0.1% sodium azide. Samples were then incubated over night at 4 °C with CD14-APC antibody (clone HCD14, no. 325608, BioLegend) and with biotinylated anti-ER Ab-1 (clone AER314, no. MS-168-B1 by Thermo Fisher Scientific) diluted in blocking buffer to a final concentration of 4 µg/ml and 2 µg/ml, respectively. After three washes with PBS-/- (for 5 min each) immunofluorescence staining was completed by incubation for 60 min at RT with AlexaFluor555-streptavidin conjugate (at 2 µg/ml, no. S-32355, Thermo Fisher Scientific) and Hoechst 33342 (at 1 µg/ml, no. H3570, Thermo Fisher) diluted in blocking buffer. After three final washing steps in PBS-/- for 5 min each, samples were mounted with Fluoromount-G (no. 0100-01, SouthernBiotech, Birmingham, AL, USA).

Samples were imaged at 800x total magnification with an LSM700 laser scanning microscope (Zeiss, Oberkochen, Germany). For analysis of nuclear and cytosolic ER localization in monocytes, CD14 positive cells were evaluated with ZEN 2 blue edition software (version 3.8.99.02000, Zeiss) using the “profile” tool: A line was drawn across the cell (plasma membrane defined by CD14 staining) including the nucleus. The median fluorescence intensity of ER staining (as quantified at 78 nm resolution) was separately determined for the nuclear versus cytosolic parts of each cell. A ratio was calculated for the median ER fluorescence intensity of the nucleus divided by the median ER fluorescence intensity of the cytosol. An average of 8 (3–11) monocytes were evaluated per patient sample to calculate mean and standard deviation (SD) of recorded ratios.

### RNA isolation of monocytes, cDNA synthesis and real-time PCR

Liquid nitrogen-cryopreserved patient PBMCs were centrifuged at 300 x g for 10 min at 4 °C and the resulting pellet was resuspended in 250 µl of PBS-/- supplemented with 0.5% BSA and 1 mM EDTA. For fast monocyte isolation, the EasySep™ Human Monocyte Enrichment Kit without CD16 Depletion (Stemcell Technologies, Vancouver, BC, Canada) was applied according to manufacturer’s recommendations. Retrieved cells were again centrifuged at 300 x g for 10 min at 4 °C and resuspended in 200 µl of PBS supplemented with 0.5% BSA. Isolated monocytes generally reached a purity of > 90%, and a mean (± SD) number of 1.7 ± 1.1 × 10^5^ cells were recovered at 37 °C for 4 h.

RNA was then isolated with Monarch^®^ Total RNA Miniprep Kit (New England Biolabs, Ipswich, MA, USA) according to the manual’s specifications for “Tissue or Leukocytes”. RNA was eluted in 30 µl of nuclease-free water and 10 µl were subjected to cDNA synthesis using High-Capacity cDNA Reverse Transcription Kit (Thermo Fisher Scientific) according to the manufacturer’s protocol. 1/40 of this reaction was applied for real-time quantitative PCR (qPCR) analysis with a 7500 Fast Real-Time PCR System (Thermo Fisher Scientific) in a total volume of 20 µl using TaqMan™ Universal PCR Master Mix (Thermo Fisher Scientific) and TaqMan™ Assays (all Thermo Fisher Scientific) designed for amplification of interleukin 1 beta (IL-1β, Hs01555410_m1), C-X-C motif chemokine ligand 8 (CXCL8 = IL-8, Hs00174103_m1), oncostatin M (OSM, Hs00171165_m1), C-C motif chemokine ligand 3 (CCL3, Hs00234142_m1), and C-C motif chemokine ligand 4 (CCL4, Hs99999148_m1). Gene expression was calculated with the 2^-∆∆Ct^ method and was normalized to beta-2-microglobulin (β2M, Hs00187842_m1).

### Histology and immunofluorescence staining of breast cancer tissue

Sections of breast cancer samples (paired biopsy specimens and surgical material) were subjected to deparaffinization with xylene and subsequent rehydration using descending alcohol concentrations. Antigen retrieval was performed by boiling slides in 10 mM TRIS, 1 mM EDTA (pH 9.0) and 0.05% Tween-20 for 20 min. Specimens were first stained for monocytes with mouse anti-human CD14 antibody (clone 5A3B11B5, final concentration 2 µg/ml, Santa Cruz, Dallas, TX, USA) over night at 4 °C, followed by detection with anti-mouse Alexa Fluor™ (AF) 488 labelled cross-adsorbed secondary antibody (45 min at RT, final concentration 5 µg/ml, Thermo Fisher Scientific). Mouse anti-human CD16 (clone DJ103c, AF647 conjugated, final concentration 5 µg/ml, Santa Cruz) and mouse anti-human estrogen receptor (ER, clone AER314, biotinylated, final concentration 2 µg/ml, Thermo Fisher Scientific) were then applied over night at 4 °C. ER was visualized by incubation with AF555-streptavidin conjugate (final concentration 2 µg/ml, Thermo Fisher Scientific) for 45 min at RT. DNA was stained with Hoechst 33342 (final concentration 10 µg/ml, Thermo Fisher Scientific) for 20 min at RT. All antibodies and detection reagents were diluted in wash buffer (20 mM TRIS, 137 mM NaCl, 0.05% Tween-20) with 1% BSA. Sections were mounted with Fluoromount-G (SouthernBiotech, Birmingham, AL, USA). Hematoxylin-eosin (HE) staining was performed according to a standard procedure using Mayer′s hemalum solution and alcoholic eosin Y solution (both Sigma-Aldrich). Sections were visualized on a Zeiss Axio Observer Z.1 microscope (Zeiss, Oberkochen, Germany) equipped with TissueFAXS software (version 7.1, build 6245.0139, TissueGnostics, Vienna, Austria) at 200x total magnification.

### Statistical analysis

Calculations were performed with statistical analysis system software (SAS^®^ version 9.3 or higher) and SPSS 26.0 (IBM). Descriptive statistics (median, range, number, percentage) are given for demographic and baseline parameters of patients according to treatment groups or response categories of RCB 0/I (≤ 1.36) versus RCB II/III (> 1.36). The distribution of investigated parameters is illustrated by boxplots, and differences between groups or over time are evaluated by non-parametric Mann-Whitney U (Wilcoxon rank sum) test or Wilcoxon signed-rank test, respectively. Fisher´s exact test or chi-square test were applied to assess group differences of categorical variables. Correlations between ratio of intermediate to classical monocytes (shifts from baseline to mid-therapy) and changes of cytokines or chemokines in patient plasma or PBMCs were measured by Spearman correlation tests.

## Results

### Study groups show comparable demographics and baseline variables

A total of 73 of the 89 BC patients (82%) in the endocrine treatment cohort of the ABCSG34 trial were included in the monocyte study. Of those, 34 women received concomitant tecemotide vaccination, while 39 women did not. Tumor biopsy samples served to determine clinical baseline characteristics. No significant differences between treatment groups were recorded in demographic or clinical parameters (Table 1). Overall, 45% of patients presented with T1 stage and 78% of cases were without lymph node involvement. None of the patients had metastatic disease. Despite the specified inclusion criteria for postmenopausal women with ER positive, Her2 negative BC of G1 or G2 grade, the study participants included 1 patient with a Her2 + tumor, 4 patients with grade 3 cancer and 1 premenopausal woman.

**Table 1:** Demographic and baseline variables of BC patients by treatment group

	With tecemotide *N* = 34	Without tecemotide *N* = 39	Total *N* = 73	*p*-value^1^
**Age (years)**				
*n* (%)	34 (100.0%)	39 (100.0%)	73 (100.0%)	.
median	65.5	68.0	68.0	.
min	52.0	52.0	52.0	.
max	81.0	81.0	81.0	.
Wilcoxon				0.397
**Body-mass index - n (%)**				
normal	9 (26.5%)	9 (23.1%)	18 (24.7%)	.
overweight	18 (52.9%)	18 (46.2%)	36 (49.3%)	.
obese	7 (20.6%)	12 (30.8%)	19 (26.0%)	.
Chi-Square				0.613
**Menopausal status - n (%)**				
postmenopausal	34 (100.0%)	38 (97.4%)	72 (98.6%)	.
premenopausal	0 (0.0%)	1 (2.6%)	1 (1.4%)	.
Fisher				1.000
**T-stage - n (%)**				
T1	16 (47.1%)	17 (43.6%)	33 (45.2%)	.
T2/T3/T4	18 (52.9%)	22 (56.4%)	40 (54.8%)	.
Chi-Square				0.766
**N-stage - n (%)**				
negative	25 (73.5%)	32 (82.1%)	57 (78.1%)	.
positive	9 (26.5%)	7 (17.9%)	16 (21.9%)	.
Chi-Square				0.380
**M-stage - n (%)**				
M0	34 (100.0%)	39 (100.0%)	73 (100.0%)	.
**Grading - n (%)**				
G1	5 (14.7%)	10 (25.6%)	15 (20.5%)	.
G2/Gx	28 (82.4%)	26 (66.7%)	54 (74.0%)	.
G3	1 (2.9%)	3 (7.7%)	4 (5.5%)	.
Fisher				0.327
**Her2 - n (%)**				
negative	30 (88.2%)	36 (92.3%)	66 (90.4%)	.
positive	1 (2.9%)	0 (0.0%)	1 (1.4%)	.
missing	3 (8.8%)	3 (7.7%)	6 (8.2%)	.
Fisher				0.463
**ER - n (%)**				
positive	34 (100.0%)	39 (100.0%)	73 (100.0%)	.
**PR - n (%)**				
negative	4 (11.8%)	3 (7.7%)	7 (9.6%)	.
positive	30 (88.2%)	35 (89.7%)	65 (89.0%)	.
missing	0 (0.0%)	1 (2.6%)	1 (1.4%)	.
Fisher				0.700
**Ki67 (%)**				
*n* (%)	34 (100.0%)	35 (89.7%)	69 (94.5%)	.
median	10	10	10	.
min	3	1	1	.
max	80	40	80	.
Wilcoxon				0.383

*ER = estrogen receptor; Her2 = human epidermal growth factor receptor 2; PR = progesterone receptor; N = number of patients in treatment groups; n = number of patients with parameter record; min = minimum; max = maximum*

^*1*^ *p-values relate to the comparison between patients with and without tecemotide as determined by Wilcoxon rank sum test for metric variables or Fisher´s exact test or chi-square test for categorical variables*

The group of healthy controls (*N* = 40) was matched for sex and age, i.e. women had a median age of 66 years (range 46–75), which was comparable to the BC patients (*N* = 73) with a median of 68 years (range 52–81).

### Breast cancer patients differ from healthy women in frequency but not in ER expression of monocyte subsets

**Fig. 2:**
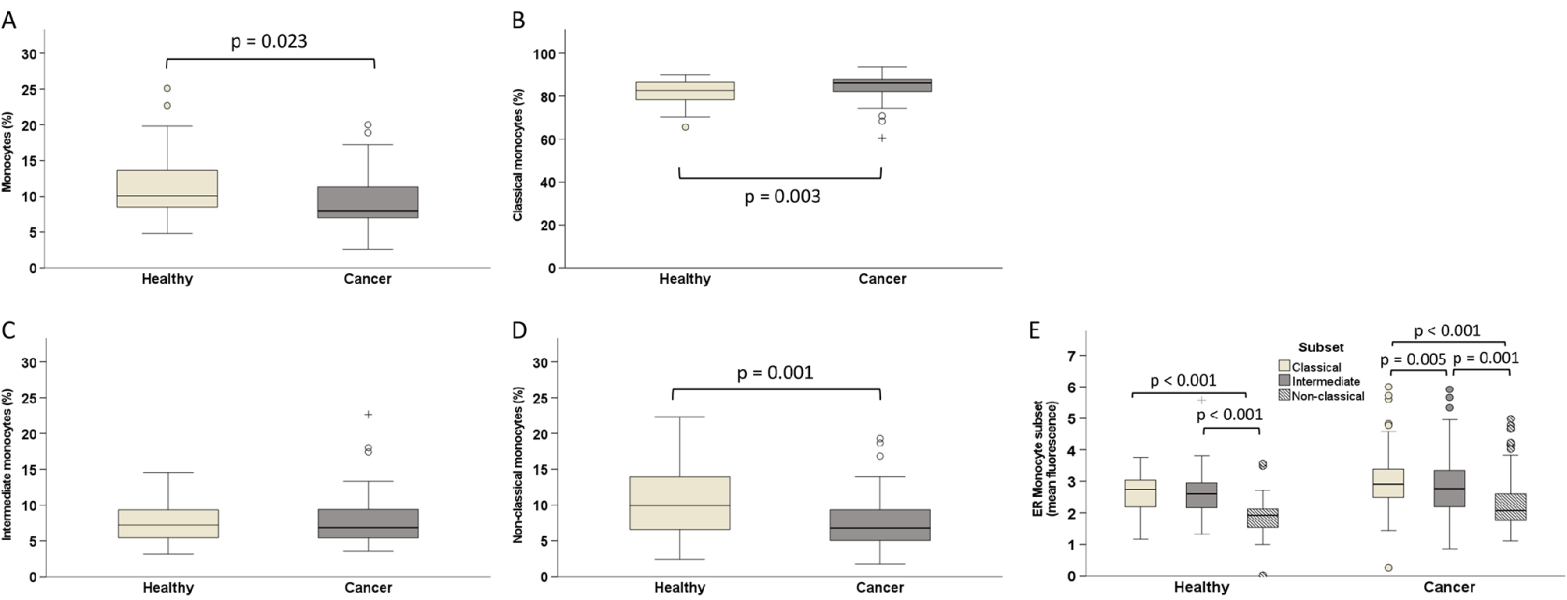
Frequency of monocyte populations as determined by flow cytometry in peripheral blood of postmenopausal BC patients compared to healthy, age-matched women. **(A)** The percentage of all monocytes (among leukocytes) as well as the frequency of subsets within the monocyte population are shown by boxplots: **(B)** classical, **(C)** intermediate, **(D)** non-classical monocytes. **(E)** Comparison of ER expression levels between monocyte subsets as separately evaluated for BC patients and healthy women. Group differences are evaluated by non-parametric Mann-Whitney U (**A**-**D**) test or Wilcoxon signed-rank test **(E)**. Circles indicate outliers with distances from the interquartile range (IQR) greater than 1.5 times the IQR, and crosses indicate extreme values with distances greater than 3 times the IQR

The percentage of monocytes among leukocytes and the frequency of the specified subsets within monocytes was evaluated by flow cytometry in BC patients before treatment start and in healthy controls. Overall, patients had lower monocyte counts (median 8%) than healthy individuals (median 10%; Fig. 2A). Furthermore, postmenopausal breast cancer patients showed an altered distribution of monocyte subsets, with more CD14 + + CD16- classical (median 86% versus 83%) and fewer CD14 + CD16 + + non-classical monocytes (7% versus 10%; Fig. 2B, D). Intermediate monocytes ranged at a comparable level of 7% in both groups (Fig. 2C).

Intracellular expression of ERα was also assessed by flow cytometry in monocytes of healthy individuals and breast cancer patients before initiation of neoadjuvant therapy. There was no difference in ER levels between the two cohorts, whether evaluated for the entire monocyte population (data not shown) or for the individual monocyte subsets. However, the three subsets differed in their ER expression, with CD14 + CD16 + + non-classical monocytes exhibiting lower levels than CD14 + + CD16 + intermediate or CD14 + + CD16- classical monocytes which had the highest ER levels (Fig. 2E).

### Endocrine therapy triggers an increase of ER levels in all monocytes while cancer vaccination shows no overall impact on monocyte parameters

**Fig. 3:**
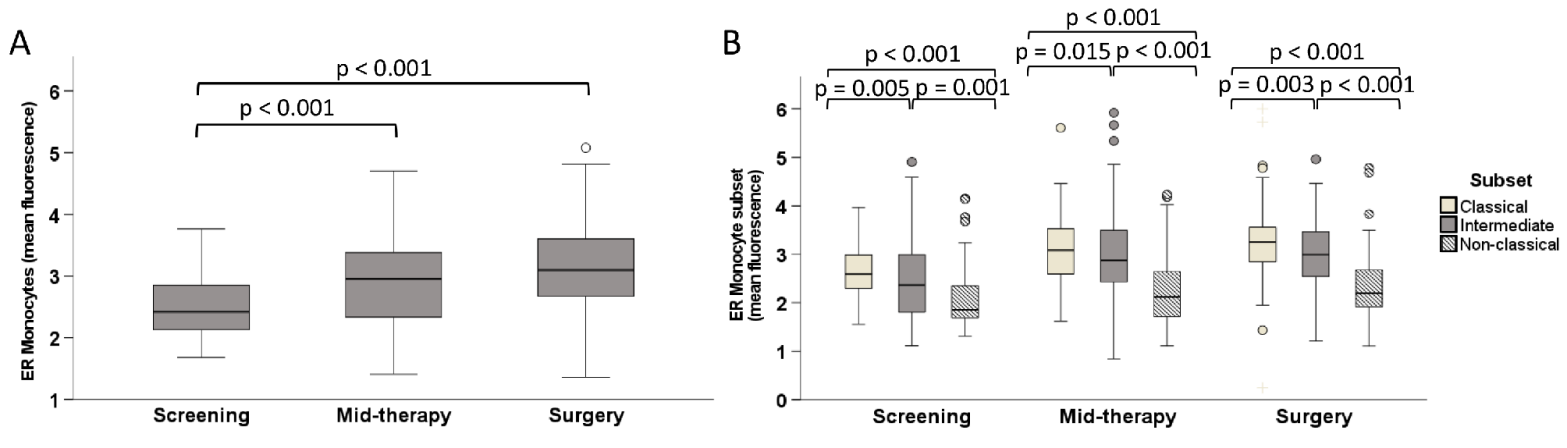
ER expression of total monocytes **(A)** and monocyte subsets **(B)** in the entire collective of 73 BC patients during neoadjuvant therapy as determined by flow cytometry at baseline (screening), after 12 weeks of letrozole administration (mid-therapy) and at the end of endocrine treatment (prior to surgery). Statistical analysis is based on Wilcoxon signed-rank test. Circles indicate outliers with distances from the interquartile range (IQR) greater than 1.5 times the IQR, and crosses indicate extreme values with distances greater than 3 times the IQR

The flow cytometric analysis of monocyte populations and the intracellular detection of ER levels was further conducted in BC patients on neoadjuvant endocrine therapy. Blood samples retrieved at baseline (during the screening phase) were compared to samples from mid-term (week 12 of letrozole therapy) and upon completion of neoadjuvant treatment (before surgery). When the entire patient population was evaluated irrespective of treatment arm, the administration of endocrine therapy had no impact on the frequency of circulating monocyte populations (Supplementary Fig. 2) but led to a significant increase in ER expression in all subsets (Fig. 3) thereby preserving the pattern of highest levels in classical and lowest levels in non-classical monocytes.

Since transcriptional ER activity is regulated by ligand (estrogen) binding and subsequent translocation from cytosol to nucleus, we further addressed the notion that endocrine therapy might also alter the intracellular localization of estrogen receptor. Yet, immunofluorescence staining of ER and confocal microscopy of PBMCs isolated from BC patients before and after neoadjuvant therapy did not differ in the intracellular ER distribution when assayed without additional in vitro stimulation (Supplementary Fig. 3).

Notably, when comparing the two treatment arms with and without tecemotide administration, vaccination did not induce a general shift among the monocyte populations. Likewise, ER expression of monocytes did not differ between treatment arms with and without cancer vaccine (Supplementary Fig. 4).

### Monocyte parameters are associated with patient response as reflected in a low residual cancer burden after neoadjuvant therapy

Treatment response based on residual cancer burden was evaluated for 71 of 73 BC patients. With the RCB cut-off set to 1.36, eleven women scored ≤ 1.36 (RCB 0/I), while 60 women showed an RCB index higher than 1.36 (RCB II/III). Demographic and baseline variables were mostly comparable between these two groups, i.e. differences were limited to a higher frequency of tumor stage T1 (*p* = 0.002) among patients with low RCB score (Table 2) in line with the notion that lower tumor stages show a more favorable therapy outcome than advanced stages.

**Table 2:** Demographic and baseline variables of BC patients by response group

	RCB ≤ 1.36 *N* = 11	RCB > 1.36 *N* = 60	Total *N* = 71	*p*-value^1^
**Age (years)**				
*n* (%)	11 (100.0%)	60 (100.0%)	71 (100.0%)	.
median	69.0	67.5	68.0	.
min	58.0	52.0	52.0	.
max	74.0	81.0	81.0	.
Wilcoxon				0.787
**Menopausal status - n (%)**				
postmenopausal	11 (100.0%)	59 (98.3%)	70 (98.6%)	.
premenopausal	0 (0.0%)	1 (1.7%)	1 (1.4%)	.
Fisher				1
**T-stage - n (%)**				
T1	10 (90.9%)	22 (36.7%)	32 (45.1%)	.
T2/T3/T4	1 (9.1%)	38 (63.3%)	39 (54.9%)	.
Fisher				0.002
**N-stage - n (%)**				
negative	9 (81.8%)	46 (76.7%)	55 (77.5%)	.
positive	2 (18.2%)	14 (23.3%)	16 (22.5%)	.
Fisher				1
**M-stage - n (%)**				
M0	11 (100.0%)	60 (100.0%)	71 (100.0%)	.
**Grading - n (%)**				
G1	4 (36.4%)	11 (18.3%)	15 (21.1%)	.
G2/Gx	7 (63.6%)	45 (75.0%)	52 (73.2%)	.
G3	0 (0.0%)	4 (6.7%)	4 (5.6%)	.
Fisher				0.318
**Her2 - n (%)**				
negative	11 (100.0%)	54 (90.0%)	65 (91.5%)	.
missing	0 (0.0%)	6 (10.0%)	6 (8.5%)	.
**ER - n (%)**				
positive	11 (100.0%)	60 (100.0%)	71 (100.0%)	.
**PR - n (%)**				
negative	0 (0.0%)	7 (11.7%)	7 (9.9%)	.
positive	11 (100.0%)	52 (86.7%)	63 (88.7%)	.
missing	0 (0.0%)	1 (1.7%)	1 (1.4%)	.
Fisher				0.587
**Ki67 (%)**				
*n* (%)	11 (100.0%)	56 (93.3%)	67 (94.4%)	.
median	10.0	10.0	10.0	.
min	5.0	1.0	1.0	.
max	30.0	80.0	80.0	.
Wilcoxon				0.567

*ER = estrogen receptor; Her2 = human epidermal growth factor receptor 2; PR = progesterone receptor; RCB = residual cancer burden index; N = number of patients in treatment groups; n = number of patients with parameter record; min = minimum; max = maximum*

^*1*^ *p-values relate to the comparison between patients with RCB index ≤ 1.36 (RCB 0/I) and patients with RCB index > 1.36 (RCB II/III) as determined by Wilcoxon rank sum test for metric variables or Fisher´s exact test or chi-square test for categorical variables*

**Fig. 4:**
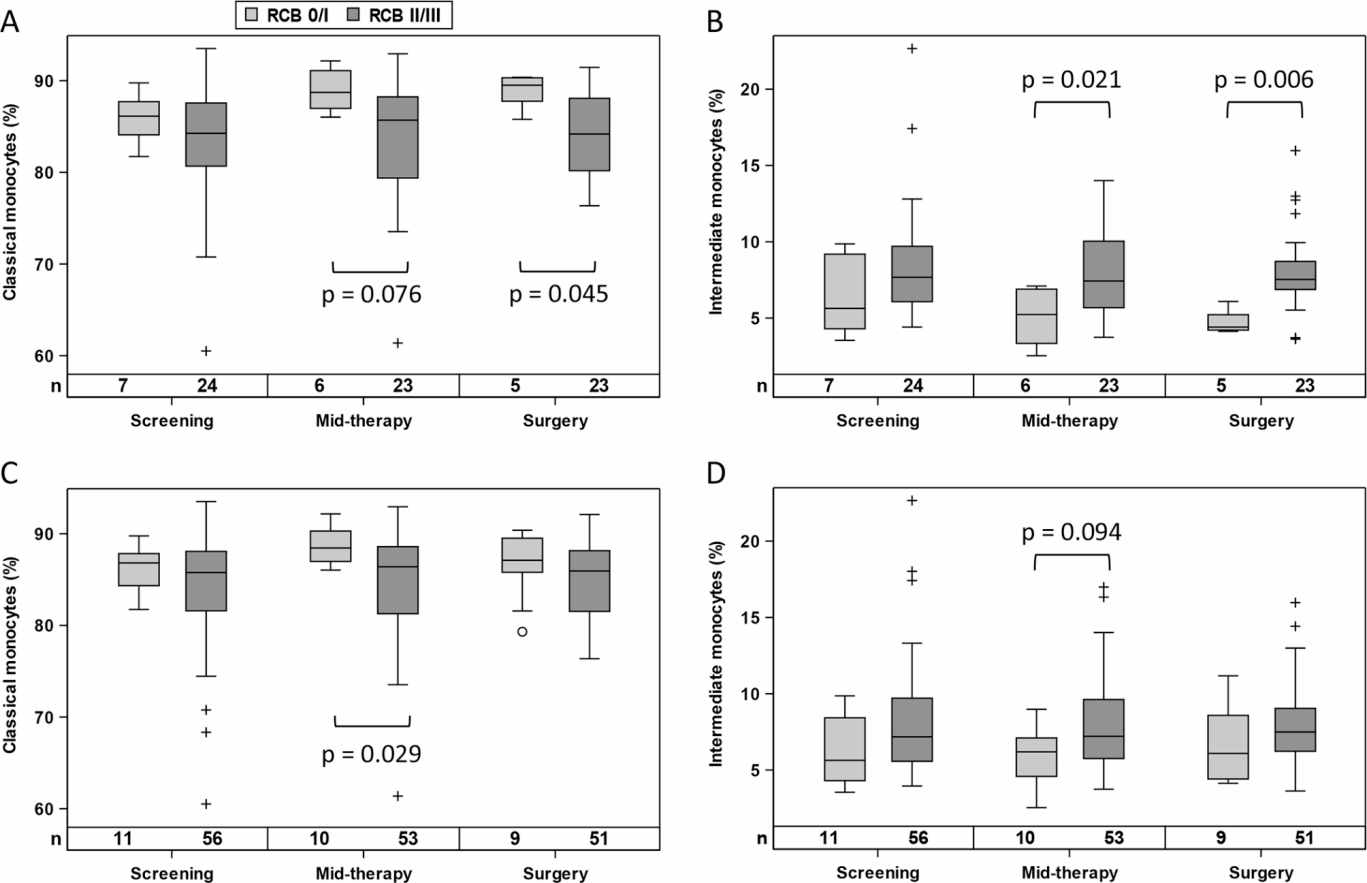
Frequency of classical (**A**, **C**) and intermediate (**B**, **D**) monocytes in patients with RCB 0/I compared to RCB II/III, when analyzed for the tecemotide treatment arm (**A**, **B**) or the entire collective (**C**, **D**). Statistical analysis is based on Mann-Whitney U test. Circles and crosses indicate data points with distances from the interquartile range (IQR) greater than 1.5 times the IQR for RCB 0/I and RCB II/III responders, respectively

**Fig. 5:**
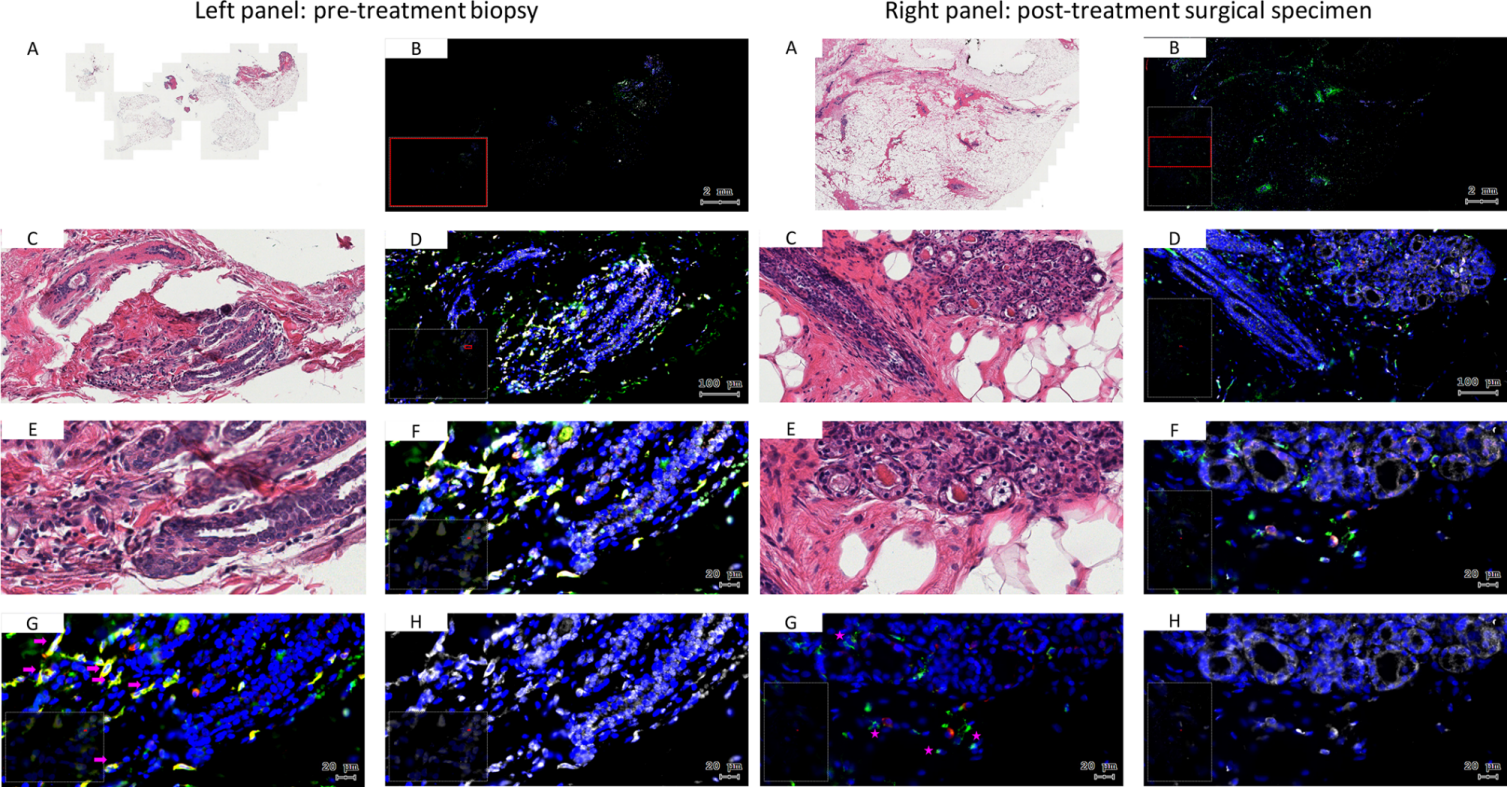
Analysis of matching breast cancer tissue retrieved by biopsy before neoadjuvant therapy (left panel) and surgically resected after treatment (right panel) from a patient with RCB 1 score and a rise in classical blood monocytes during neoadjuvant therapy. Whole tissue scans (**A**, **B**) and zoom-in regions (**C**-**H**) are shown of H&E stained sections (**A**, **C**, **E**) or immunofluorescence stainings (**B**, **D**, **F**) of CD14 (green), CD16 (red), ER (white) and DNA/cell nuclei (blue). For better resolution of monocyte subsets and ER expression, the total color overlay of image **F** is further split into CD14 (green), CD16 (red) in image **G** and ER (white) in image **H**. Pink arrows indicate CD14 + CD16 + monocytes (yellow color overlay of red and green), while pink asterisks identify classical CD14 + CD16- monocytes (green)

Monocyte subsets and their ER expression levels were further evaluated with respect to treatment response. Patients exhibiting a positive therapy outcome (RCB 0/I) showed a shift from CD14 + + CD16 + intermediate monocytes to CD14 + CD16- classical monocytes at mid-therapy and pre-surgery, which was particularly evident in the tecemotide treatment arm (Fig. 4A-B) but was also observed in the overall population (Fig. 4C-D). To ascertain whether the respective monocyte populations were also detected in breast cancer tissue, we stained pre-treatment biopsies and surgery specimens from selected patients. Before therapy the majority of infiltrating monocytes were double positive for CD14 and CD16, while the resected post-treatment RCB1 sample had a higher prevalence of CD16 negative classical monocytes (Fig. 5) which was not the case for an RCB 2 patient who had no rise in classical blood monocytes (Supplementary Fig. 5).

**Fig. 6:**
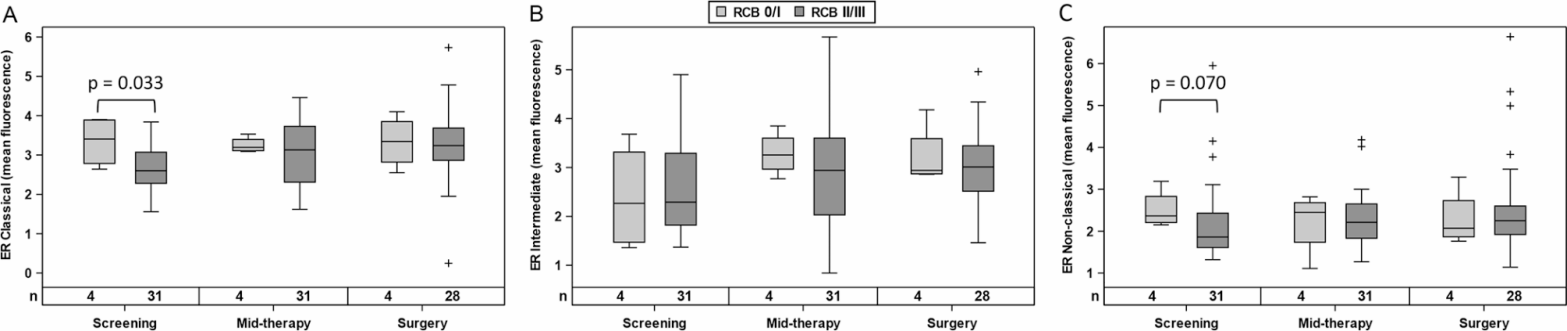
ER expression level of classical **(A)**, intermediate **(B)** and non-classical **(C)** monocytes in patients with RCB 0/I compared to RCB II/III, when analyzed for the endocrine treatment arm without cancer vaccination. Group differences are evaluated by Mann-Whitney U test. Circles and crosses indicate data points with distances from the interquartile range (IQR) greater than 1.5 times the IQR for RCB 0/I and RCB II/III responders, respectively

In patients without tecemotide, baseline levels of ER expression in classical monocytes were higher in good responders to endocrine therapy (Fig. 6A) with a similar trend in non-classical monocytes (Fig. 6C) but not in intermediate monocytes (Fig. 6B). ER expression levels did not show any association with RCB score for BC patients with additional tumor vaccination or when the combined treatment groups were evaluated (data not shown).

### A shift of monocyte subsets upon cancer vaccination is associated with concomitant changes in plasma cytokines and chemokines

For a limited number of patients (7 women with and 8 women without tecemotide therapy) the plasma samples collected at baseline and mid-therapy were investigated by multiplex immunoassay for 92 circulating human cytokines and chemokines. With respect to the shift from intermediate to classical monocytes that was associated with treatment response in the tecemotide arm, we found changes in 13 plasma parameters which showed a high correlation (*r* ≥ 0.8 or ≤ -0.8) with the monocyte alterations (Table 3). Cytokines with a direct (positive) correlation comprised oncostatin M (OSM), interleukin (IL) 6, IL-8 and macrophage inflammatory protein 1α (CCL3) and 1β (CCL4). A negative association was recorded for IL-4, IL-18 receptor 1, IL-20 and IL-20 receptor alpha, monocyte chemoattractant protein-1 (MCP-1, CCL2), C-C chemokine ligand 23 (CCL23), leukemia inhibitory factor (LIF) receptor and urokinase plasminogen activator (uPA). In contrast, no significant correlations (*r* ≥ 0.8 or ≤ -0.8) were recorded for circulating cytokines and baseline levels of ER expression in classical monocytes which held predictive information for response to endocrine therapy in patients without tecemotide.

**Table 3:** Correlation between the ratio of intermediate to classical monocytes (shift from baseline to mid-therapy) and the detected changes of cytokines or chemokines in plasma of patients treated with tecemotide

Plasma parameter	Coefficient of correlation *r*^1^	*p*-value^1^
Interleukin 4	-0.802	0.030
Interleukin 6	0.857	0.014
Oncostatin M	0.857	0.014
Leukemia inhibitory factor receptor	-0.893	0.007
Interleukin 8	0.857	0.014
Interleukin 18 receptor 1	-0.964	< 0.001
Interleukin 20	-0.802	0.030
Interleukin 20 receptor subunit alpha	-0.802	0.030
Macrophage inflammatory protein 1α	0.857	0.014
Macrophage inflammatory protein 1β	0.857	0.014
Monocyte chemoattractant protein-1	-0.964	< 0.001
C-C chemokine ligand 23	-0.821	0.023
Urokinase plasminogen activator	-0.893	0.007

*The subset ratio was calculated by dividing the percentage of classical monocytes by the frequency of intermediate monocytes; the ratio determined at baseline was subtracted from the ratio established at mid-therapy. Comparably*,* the change in plasma cytokine levels from screening to mid-term was calculated and analyzed for potential correlation (r ≥ 0.8 or ≤ -0.8) with the shift in monocyte subsets*

^*1*^ *Coefficients of correlation r and p-values relate to analysis by Spearman correlation test*

To address the question whether the detected plasma concentrations of cytokines and chemokines were related to gene expression by circulating monocytes in patients, we isolated monocytes from 9 available corresponding PBMC samples for qPCR analysis of selected transcripts (IL-6, IL-1β, IL-8, TNFα, OSM, CCL2, CCL3, CCL4, CCL23). While monocyte purity was high (> 90%), mRNA levels were below detection limit for 5 patients and for IL-6, CCL2 and CCL23 transcripts. Baseline and mid-term samples of the remaining patients yielded the following Spearman coefficients of correlation between monocyte transcript and plasma protein levels: IL-1β (*n* = 6): 0.507, IL-8 (*n* = 7): 0.357, OSM (*n* = 7): 0.464, CCL3 (*n* = 7): 0.679 and CCL4 (*n* = 8): 0.443.

## Discussion

Based on the notion that monocytes may be a target of endocrine therapy [30] and of the cancer vaccine tecemotide [19, 20], we investigated the subset composition and ER expression of circulating monocytes in BC patients in the ABCSG34 trial. When we initially compared the pre-treatment levels of patients with sex- and age-matched healthy controls, women with BC exhibited an overall lower monocyte count and an increased frequency of the classical versus non-classical monocyte subset. These findings differ from previous studies where a higher frequency of CD16 + monocytes was reported for BC patients versus matched healthy controls which was negatively associated with tumor size and pathological stage [39, 40]. Other studies confirmed increased blood levels for intermediate [41] and non-classical monocytes [42] in women with BC. Of note, the non-classical monocytes presented with reduced expression of TNFα [43] and high levels of the immune checkpoint molecule programmed death-ligand 1 [44], thus indicating an immunosuppressive phenotype. The elevated frequency of classical monocytes which we detected in BC patients of our study is in stark contrast to these previous findings and might relate to the included cancer subtype. While our investigation focused on postmenopausal women with non-metastasized, Her2-, ER + BC, other studies generally included pre- and postmenopausal women of all BC subtypes and tumor stages [39–41, 43]. Since tumors are proposed to direct the systemic reprogramming of monocytes and their subset composition [45], our data might indicate that circulating monocytes are shaped by early stage ER + Her2- breast carcinoma in a manner distinct from other BC subtypes and/or later stages. Interestingly, estrogen was reported to negatively regulate CD16 expression which was reversed by fulvestrant [46, 47]. While this supports the observed high levels of CD16- classical monocytes at baseline (and highest ER expression levels in classical monocytes), it would also suggest an increase in the CD16 + subsets upon endocrine therapy which was, however, not apparent.

In contrast, AI treatment resulted in a detectable rise in ER expression in all monocyte subsets over time. This upregulation in receptor levels is likely a feedback response to the decreased blood levels of estrogen to regulate cellular sensitivity to estrogen signals [48]. Yet, it did not alter ER distribution between cytosol and nucleus as illustrated by immunostaining and confocal microscopy of patient monocytes (without additional in vitro stimulation). Of note, we cannot exclude that the in vivo pretreated monocytes might react differently with respect to intracellular ER localization if tested in a second in vitro challenge by estrogen or other stimuli. Primary human monocytes were reported to express different ER variants: Both, ERα and ERβ were constitutively present at transcript and protein level [22, 49]. With respect to ERα splice variants, not only the wildtype (full-length) ERα66 was detected but also shorter isoforms termed ERα46 [50] and ERα36 as well as the interacting G-protein estrogen receptor 1 [51]. Of note, the antibody applied for ER detection in our study (clone AER314) is expected to detect the wildtype ERα66 protein but not likely to bind the smaller splice variants. Thus, the reported regulation and biomarker value in this study relates to the full-length ERα receptor, while changes in other ER variants cannot be deduced.

With respect to treatment response, BC patients were grouped into low (RCB 0/I) versus high (RCB II/III) residual cancer burden. Regarding the predictive potential of monocyte parameters for neoadjuvant therapy, a higher baseline level of ERα expression (in particular in the classical monocytes) was associated with a better treatment response to endocrine therapy. This association was not observed when endocrine therapy was combined with cancer vaccination. In contrast, the tecemotide treatment arm showed a change in monocyte subset frequency in good responders. Patients with RCB 0/I tended to accumulate more circulating classical and fewer intermediate monocytes in the course of neoadjuvant therapy which was also reflected in corresponding breast cancer tissue (in a limited sample set investigated in a qualitative manner). The change in circulating monocyte subset distribution was already detectable at mid-term and may reflect the reduced tumor capacity to reprogram circulating monocytes to the tumor-promoting CD16 + subsets [45]. An increase in classical monocytes was also reported for BC patients (ER + or Her2+) under neoadjuvant chemotherapy with doxorubicin and cyclophosphamide [52]. However, these authors did not detect an association between levels of classical monocytes and pathological complete response in ER + BC patients which may be due to the different combinations of therapeutic agents (not involving endocrine therapy or cancer vaccination).

To address the potential immunological implications of monocyte shifts to the inflammatory classical phenotype as observed for responders of the tecemotide arm, the concomitant changes in plasma cytokines were investigated in a subset of patients. The remarkably strong negative association of CCL2 (*r*=-0.964) was in good agreement with an earlier study which demonstrated the predominant role of CCL2 in sustaining the intermediate monocyte phenotype in breast cancer patients [40]. Other parameters that were inversely correlated with the shift to classical monocytes, i.e. which were concomitantly decreased, comprised CCL23 and uPA which have previously been reported as biomarkers for poor prognosis in BC patients [53, 54]. Furthermore, the negative regulation of IL-4 and the positive correlation of CCL3, CCL4 indicate a shift from M2 towards M1 macrophage polarization [55, 56] which would support the notion of therapy response. Also the decrease in IL-20 might contribute to treatment efficacy, since anti-IL-20 antibodies suppress breast cancer progression in mice [57]. In contrast, IL-8 and members of the IL-6 cytokine family (IL-6, OSM and LIF) are proposed to drive BC proliferation, invasion and metastasis [58, 59]. The observed associations with monocyte shifts would hence be expected to worsen rather than improve patient prognosis, thus cautioning that all these findings will have to be reproduced in a larger data set and investigated for causal relations in an experimental setting. Of note, we also compared transcript levels in PBMCs with plasma concentrations of the corresponding cytokines in a limited number of matching patient samples and found a moderate correlation for IL-1β, OSM, CCL3 and CCL4, indicating that these proteins might (in part) be produced by the monocytes themselves. Yet, other factors regulating monocyte differentiation or function likely originate from sources such as the tumor and its microenvironment.

## Conclusions and limitations

Monocytes are subject to regulation by cancer therapies. While preclinical studies support their functional involvement, this study has characterized monocyte changes under neoadjuvant BC treatment and found associations of patient response with subset distribution and ER expression of monocytes. Due to the limited number of good responders when treatment arms were evaluated separately, the provided p-values have to be viewed with caution, and data validation in further studies is warranted. Furthermore, this trial was based on a well-defined patient cohort, aimed to comprise only postmenopausal women with early-stage BC of an ER + and Her2- subtype. However, in the course of the ABCSG34 trial, 1 of the 73 initially included patients was later categorized with a Her2 + tumor, and for 6 women Her2 status was not ascertained. Furthermore, 1 patient was subsequently identified as premenopausal. Nonetheless, the majority of the included participants were in agreement with the specified criteria, yielding a patient collective with 100% ER+, > 90% Her2-, and 100% non-metastasized tumors. This highly uniform patient cohort is an exceptional quality compared to most previous studies and may hence explain the interesting novel and in part differing results obtained.

## Electronic supplementary material

Below is the link to the electronic supplementary material.

Supplementary Material 1

## Data Availability

The clinical data can be shared upon approval of the analysis proposal by the Steering Committee and sponsor of the ABCSG34 study, and after a data sharing agreement has been signed. Please contact the corresponding author for more information.

## Abbreviations

ABCSG: Austrian Breast & Colorectal Cancer Study Group
AF: Alexa Fluor™
BC: Breast cancer
BSA: Bovine serum albumin
CCL: C-C chemokine ligand
CCR: C-C chemokine receptor
CPT: Cell preparation tube
CTAD: Citrate, theophylline, adenosine, dipyridamole
E2: 17β-estradiol
EDTA: Ethylenediamine tetraacetic acid
ER: Estrogen receptor
FBS: Fetal bovine serum
HE: Hematoxylin-eosin
Her2: Human epidermal growth factor receptor 2
IL: Interleukin
LIF: Leukemia inhibitory factor
MCP-1: Monocyte chemoattractant protein-1
MIP: Macrophage inflammatory protein
MUC1: Mucin-1
OSM: Oncostatin M
PBMC: Peripheral blood mononuclear cell
PBS: Phosphate-buffered saline
PR: Progesterone receptor
qPCR: Quantitative polymerase chain reaction
RCB: Residual cancer burden
TNFα: Tumor necrosis factor alpha
uPA: Urokinase plasminogen activator
