# Supplementary material for: Monocyte subsets in breast cancer patients under treatment with aromatase inhibitor and mucin-1 cancer vaccine
Source: J Transl Med. 2024 Oct 8;22:913. doi: 10.1186/s12967-024-05659-w (PMC11460172; doi:10.1186/s12967-024-05659-w)
Supplement: Supplementary file 1 — Supplementary Material 1 [file 12967_2024_5659_MOESM1_ESM.docx]

Monocyte subsets in breast cancer patients under treatment with aromatase inhibitor and mucin-1 cancer vaccine

**Viktoria Knöbl^1^, Lukas Maier^1^, Stefan Grasl^1^, Carmen Kratzer^1^, Felix Winkler^1^, Vanessa Eder^1^, Hubert Hayden^1^, Maria Amparo Sahagun Cortez^1^, Monika Sachet^2^, Rudolf Oehler^2,3^, Sophie Frantal^4^, Christian Fesl^4^, Karin Zehetner^4^, Georg Pfeiler^4,5^, Rupert Bartsch^4,6^, Florian Fitzal^2,4,7^, Christian F. Singer^4,5^, Martin Filipits^3,4,8^ , Michael Gnant^3,4^ and Christine Brostjan^1*^**

^1^Division of Vascular Surgery, Department of General Surgery, Medical University of Vienna and University Hospital Vienna, Vienna, Austria

^2^Division of Visceral Surgery, Department of General Surgery, Medical University of Vienna and University Hospital Vienna, Vienna, Austria

^3^Comprehensive Cancer Center, Medical University of Vienna, Vienna, Austria

^4^Austrian Breast & Colorectal Cancer Study Group (ABCSG), Vienna, Austria

^5^Department of Obstetrics and Gynecology, Medical University of Vienna and University Hospital Vienna, Vienna, Austria

^6^Division of Oncology, Department of Medicine I, Medical University of Vienna, Vienna, Austria

^7^Department of General Surgery, Hanusch Hospital, Vienna, Austria

^8^Center for Cancer Research, Medical University of Vienna, Vienna, Austria

*** Correspondence:**christine.brostjan@meduniwien.ac.at

**Supplementary Figures**


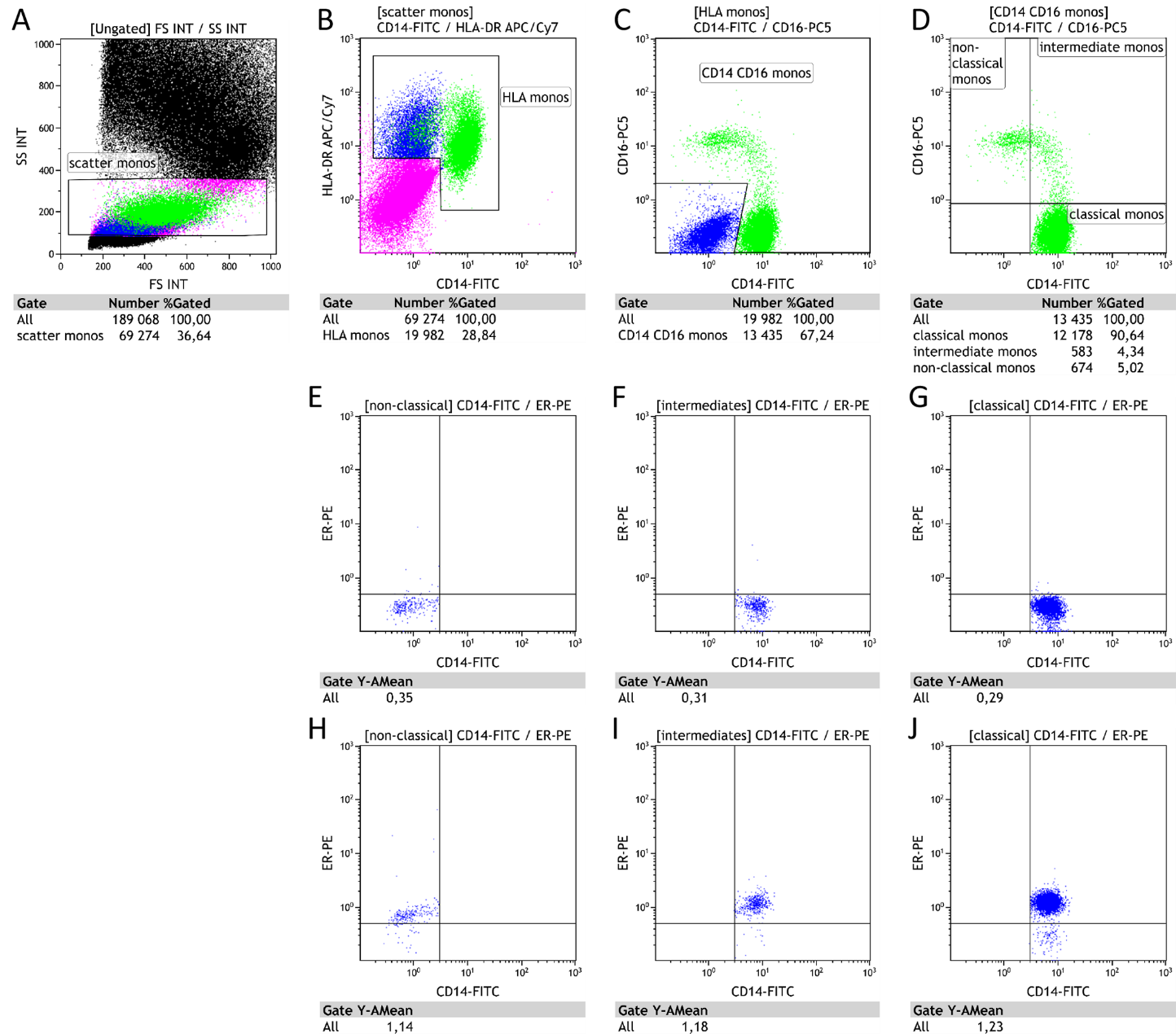


Suppl. Figure 1. Gating strategy for flow cytometric analysis of circulating monocyte populations in immunostained blood samples. (A) Monocytes were first recognized by their forward scatter (FS) and side scatter (SS) properties of laser light and (B) were then detected by fluorescence-conjugated antibodies binding to surface CD14 and HLA-DR. (C) Remaining contaminating lymphocytes were excluded by gating on CD14 and CD16 expressing cells. (D) The identified monocyte population was then further divided into classical (CD14++CD16-), intermediate (CD14++CD16+) and non-classical (CD14+CD16++) monocytes. Permeabilized cell samples were further investigated for intracellular levels of (H-J) ER by biotin-labeled antibody compared to (E-G) biotin-labeled isotype control and detection by streptavidin-PE conjugate. In addition to the (D) distribution of monocyte subsets (% Gated), their (H-J) ER expression level was recorded based on mean fluorescence intensity (Mean).


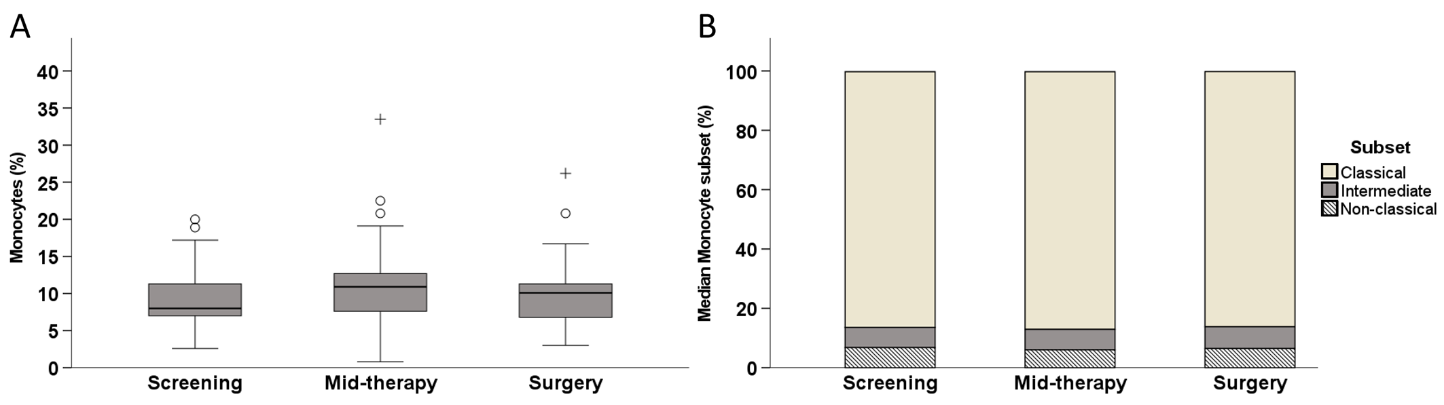


Suppl. Figure 2. Frequency of total monocytes (A) and monocyte subsets (B) in the entire collective of 73 BC patients during neoadjuvant therapy as determined by flow cytometry at baseline (screening), after 12 weeks of letrozole administration (mid-therapy) and at the end of endocrine treatment (prior to surgery). Statistical analysis is based on Wilcoxon signed-rank test. Circles indicate outliers with distances from the interquartile range (IQR) greater than 1.5 times the IQR, and crosses indicate extreme values with distances greater than 3 times the IQR.


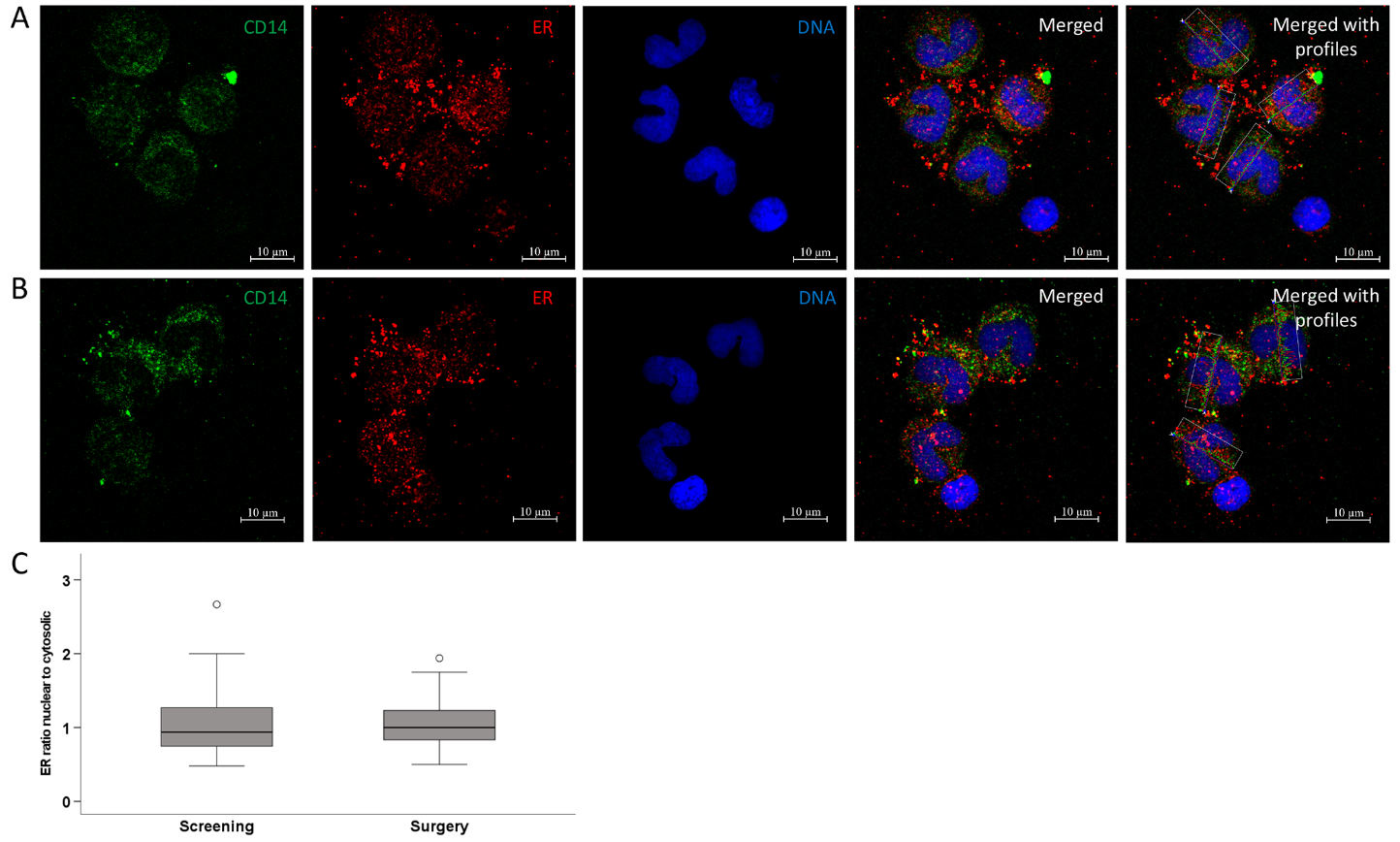


Suppl. Figure 3. Assessment of nuclear and cytosolic ER localization before and after neoadjuvant therapy of BC patients. PBMCs were isolated at (A) baseline and (B) prior to surgery and were immunostained for monocytes (CD14, green), ER (red) and DNA (blue) for confocal microscopy. A profiling tool was used to determine the intracellular ER distribution, i.e. to measure the mean ER fluorescence intensity in the cytosol versus nucleus. (C) Boxplots illustrate the ratio of nuclear to cytosolic ER as determined for 60 cells (n = 31 at screening and n = 29 at surgery) from four patients with endocrine therapy and tecemotide (p = 0.711, Wilcoxon signed-rank test). Circles indicate data points with distances from the interquartile range (IQR) greater than 1.5 times the IQR.


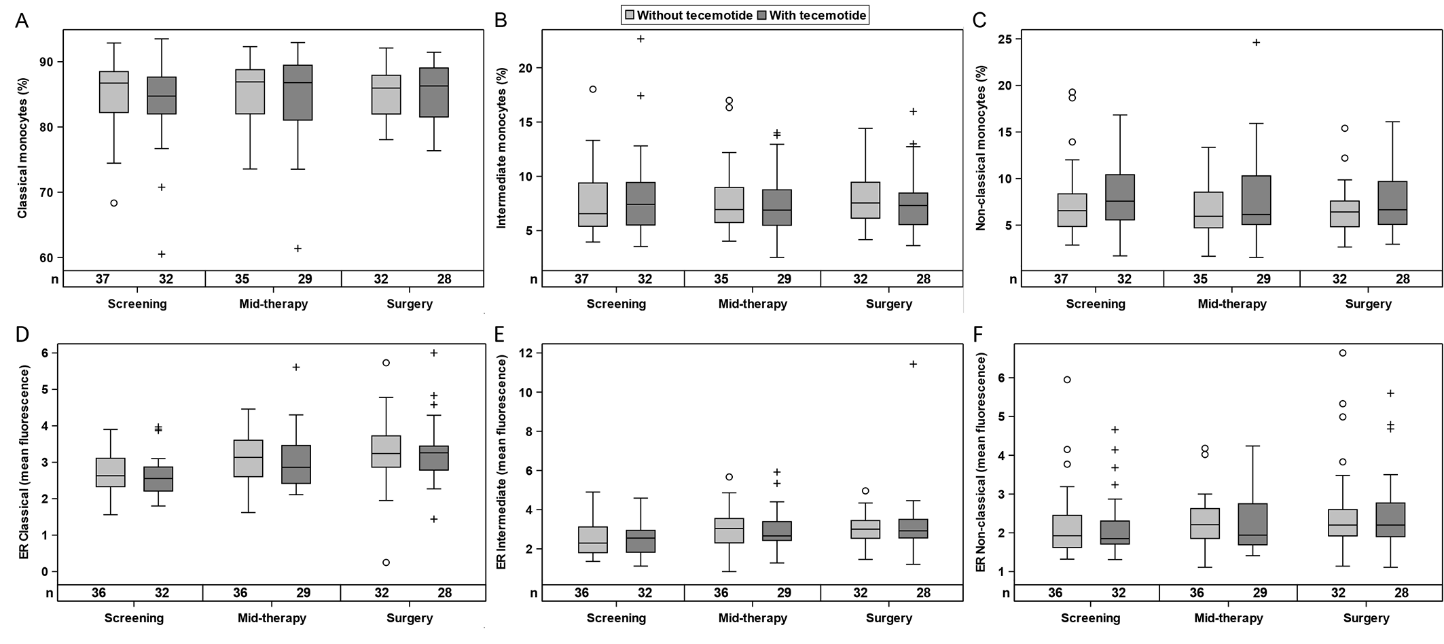


Suppl. Figure 4. Frequency (A-C) and ER expression (D-F) of classical (A,D), intermediate (B,E) and non-classical monocyte subsets (C,F) in patients with or without tecemotide vaccination. Group differences are evaluated by Mann-Whitney U test. Circles and crosses indicate data points with distances from the interquartile range (IQR) greater than 1.5 times the IQR for patients without or with tecemotide treatment, respectively.


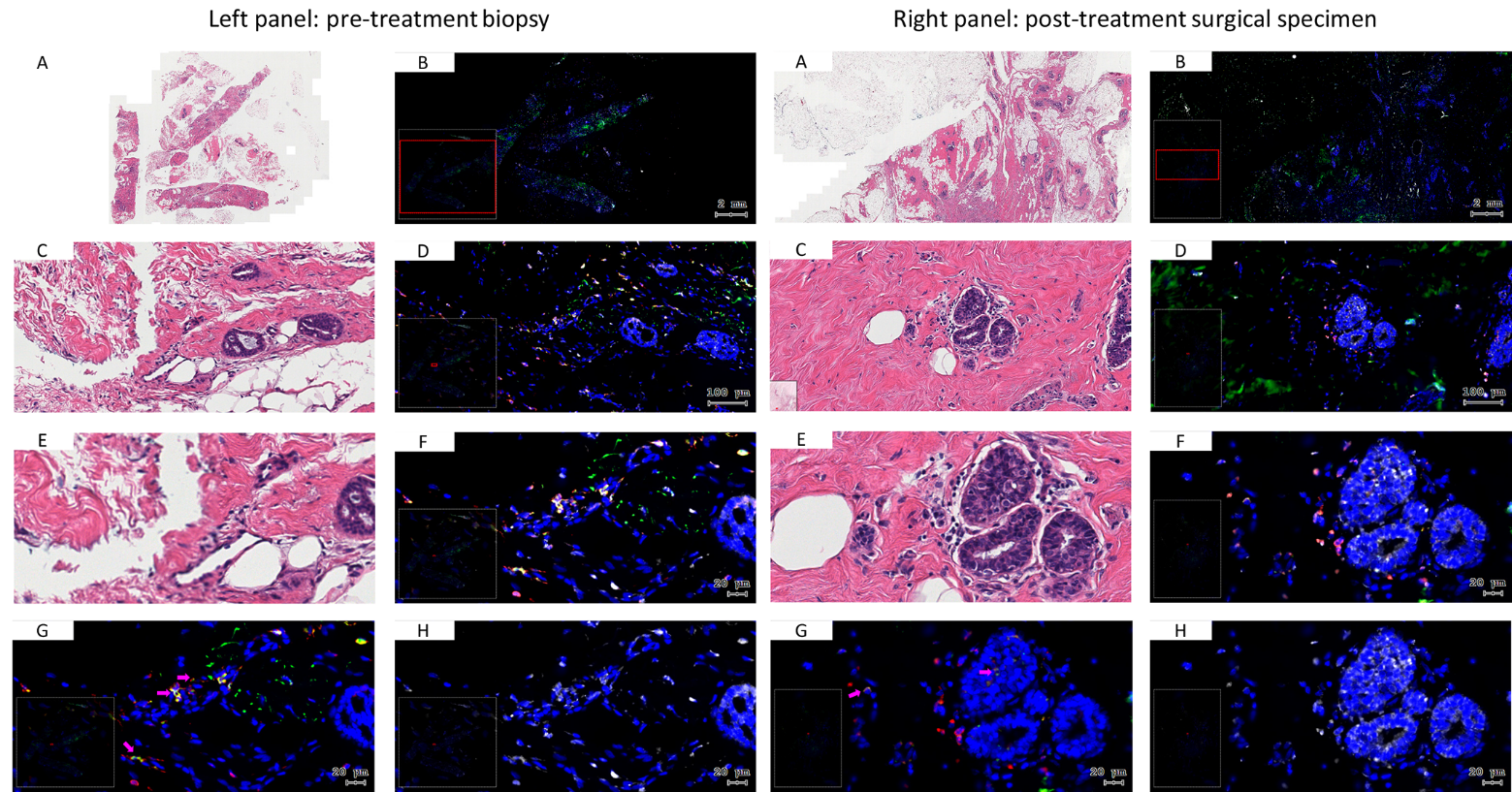


Suppl. Figure 5. Analysis of matching breast cancer tissue retrieved by biopsy before neoadjuvant therapy (left panel) and surgically resected after treatment (right panel) from a patient with RCB 2 score and without rise in classical blood monocytes during neoadjuvant therapy. Whole tissue scans (A,B) and zoom-in regions (C-H) are shown of H&E stained sections (A,C,E) or immunofluorescence stainings (B,D,F) of CD14 (green), CD16 (red), ER (white) and DNA/cell nuclei (blue). For better resolution of monocyte subsets and ER expression, the total color overlay of (F) is further split into CD14 (green), CD16 (red) in (G) and ER (white) in (H). Pink arrows indicate CD14+ CD16+ monocytes (yellow color overlay of red and green).
